# Increased Type I interferon signaling and brain endothelial barrier dysfunction in an experimental model of Alzheimer’s disease

**DOI:** 10.1038/s41598-022-20889-y

**Published:** 2022-10-01

**Authors:** Arundhati Jana, Xinge Wang, Joseph W. Leasure, Lissette Magana, Li Wang, Young-Mee Kim, Hemraj Dodiya, Peter T. Toth, Sangram S. Sisodia, Jalees Rehman

**Affiliations:** 1 Division of Cardiology, Department of Medicine, College of Medicine, University of Illinois, Chicago, IL 60612 USA; 2 Department of Biomedical Engineering, University of Illinois at Chicago, Chicago, IL 60612 USA; 3 Department of Biochemistry and Molecular Genetics, University of Illinois, College of Medicine, Chicago, IL 60607 USA; 4 Department of Neurobiology, University of Chicago, Chicago, IL 60637 USA; 5 The Microbiome Center, University of Chicago, Chicago, IL 60637 USA; 6 Research Resources Center, University of Chicago, Chicago, IL 60612 USA; 7 Department of Pharmacology and Regenerative Medicine, University of Chicago, Chicago, IL 60612 USA

**Keywords:** Physiology, Neuroscience

## Abstract

Blood–brain barrier (BBB) dysfunction is emerging as a key pathogenic factor in the progression of Alzheimer’s disease (AD), where increased microvascular endothelial permeability has been proposed to play an important role. However, the molecular mechanisms leading to increased brain microvascular permeability in AD are not fully understood. We studied brain endothelial permeability in female APPswe/PS1∆E9 (APP/PS1) mice which constitute a transgenic mouse model of amyloid-beta (Aβ) amyloidosis and found that permeability increases with aging in the areas showing the greatest amyloid plaque deposition. We performed an unbiased bulk RNA-sequencing analysis of brain endothelial cells (BECs) in female APP/PS1 transgenic mice. We observed that upregulation of interferon signaling gene expression pathways in BECs was among the most prominent transcriptomic signatures in the brain endothelium. Immunofluorescence analysis of isolated BECs from female APP/PS1 mice demonstrated higher levels of the Type I interferon-stimulated gene IFIT2. Immunoblotting of APP/PS1 BECs showed downregulation of the adherens junction protein VE-cadherin. Stimulation of human brain endothelial cells with interferon-β decreased the levels of the adherens junction protein VE-cadherin as well as tight junction proteins Occludin and Claudin-5 and increased barrier leakiness. Depletion of the Type I interferon receptor in human brain endothelial cells prevented interferon-β-induced VE-cadherin downregulation and restored endothelial barrier integrity. Our study suggests that Type I interferon signaling contributes to brain endothelial dysfunction in AD.

## Introduction

Alzheimer’s disease (AD) is a progressive neurodegenerative disorder characterized by extracellular depositions of amyloid-beta (Aβ) peptides in the brain parenchyma as senile plaques^1,2^. AD is also characterized by deposition of intracellular hyperphosphorylated tau protein in neurons leading to neuronal loss and glial activation^3^. Previous studies have suggested that Aβ and tau lead to vascular abnormalities using experimental animal models of AD^4–7^. Dysfunction of the blood–brain barrier (BBB) results in increased leakiness of the brain endothelial barrier, impairs neuronal function, and promotes neurodegeneration^1^. MRI studies in AD patients suggest that cerebral blood flow (CBF) reduction and increased BBB dysfunction in the hippocampus and cortical regions are early pathological events in AD^8,9^. Collectively, these studies indicate that increased BBB dysfunction, as manifested by increased brain endothelial barrier permeability, may be an early pathogenic process in AD. BBB breakdown in AD is associated with pathological changes of the brain microvasculature such as decreased pericyte coverage, increased soluble platelet-derived growth factor receptor-β in the cerebrospinal fluid^10,11^, increased Aβ accumulation along brain blood vessels^12^, decreased tight junction (TJ) and adherens junction (AJ) proteins^13^, transporter dysfunction^14–17^ and increased string vessel formation^18^. The pathogenic roles and mechanisms of pericyte dysfunction in AD progression have been established^11,19–21^. In this regard, while it is well established that brain endothelial cells (BECs), the cells that line the brain microvasculature regulate the integrity and permeability of the brain microvasculature by limiting the entry of toxins, pathogens, and immune cells^22,23^, the pathogenic roles of BECs and their dysfunction are less well defined.

Together with Aβ amyloidosis, preclinical and clinical studies have shown that neuroinflammation plays a key role in the pathogenesis and progression of AD^24–28^. Neuroinflammation in AD involves increased activation of microglia and astrocytes, leading to increased proinflammatory cytokine burden^29–35^. Furthermore, studies also suggest that inflammatory activation in the brain contributes to increase barrier leakiness^36–38^. To understand the mechanisms underlying the loss of brain endothelial integrity in AD, we used APP/PS1 mice that ubiquitously express a chimeric mouse/human amyloid precursor protein with the familial AD (FAD)-linked “Swedish” mutation (Mo/HuAPP695swe) and FAD-linked mutant human presenilin 1 (PS1DE9) leading to age-dependent accumulation of Aβ peptides in amyloid plaques in the hippocampus and neocortex^39–41^. In this study we focused on female APP/PS1 mice because previous reports have shown that female mice in this experimental model exhibited increased Aβ burden, tau pathology, and neuroinflammation which resulted to neuronal loss^42,43^ when compared with age-matched APP/PS1 male mice.

We observed increased brain endothelial vascular permeability in APP/PS1 female mice when compared to age-matched female control mice. Transcriptomic analysis of brain endothelial cells isolated from female APP/PS1 mice demonstrated significantly upregulated expression of interferon response genes. Moreover, exposure of a human brain endothelial cell line to Type I interferon-β (IFN-β) decreased the levels of the adherens junction protein VE-cadherin and the tight junction proteins Occludin and Claudin-5. The current results suggest that increased type I interferon signaling may promote brain endothelial barrier leakiness in a neurodegenerative disease such as Alzheimer’s disease.

## Results

### Increased blood–brain barrier leakiness in APP/PS1 mice

**Figure 1:**
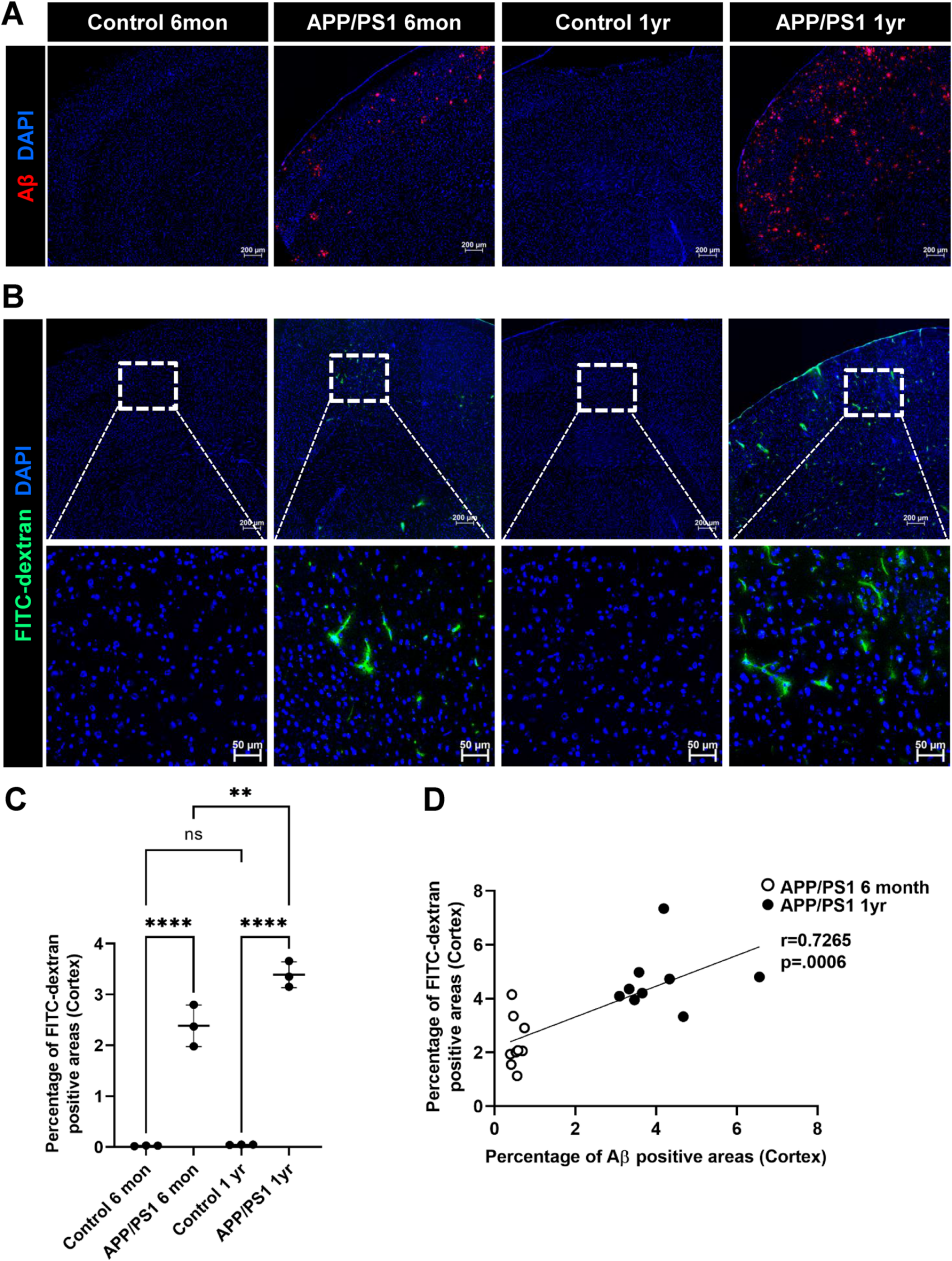
Blood–brain barrier leakiness and Aβ deposition in the cortex of APP/PS1 female mice. Control female age-matched and APP/PS1 female mice were analyzed at ages of 6 months and 1 year old for amyloid plaque by staining with antibody against Aβ (red) and BBB permeability was assessed by FITC-dextran leakage (green) in the brain parenchyma. Low-magnification representative images of coronal sections of the brain cortex for Aβ (**A**) and of FITC-dextran leakage (**B**). The ROI is outlined with a white box and subsequent images are given at higher magnification of tissue corresponding to the ROIs for controls and transgenic AD mice. Nuclei were counterstained using DAPI. Experiments were repeated at least three times with similar results. The most BBB leakage is observed in the 1 year old APP/PS1 female mice. Scale bars, 200 μm and 50 μm respectively. (**C**) Quantification of FITC-dextran leakage in the cortex of the above groups. A significant increase in BBB permeability occurs in 1 year old APP/PS1 female mice compared to 6-month-old transgenic mice. Data are represented as means and SD for three mice for each group and analyzed by one-way ANOVA followed by Tukey’s multiple comparisons test. **p = 0.0041, ****p = 0.0001, *ns* non-significant. (**D**) Correlation of Aβ deposition and FITC-dextran leak in 6 months old and 1 year old APP/PS1 female mice. FITC-dextran leakage was positively correlated with age-dependent Aβ accumulation in the cortex. Correlation coefficients and p value are indicated. *r* Pearson’s coefficient; *p* significance.

We investigated blood–brain barrier integrity and Aβ deposition in 6-month-old female APP/PS1 mice which demonstrated minimal Aβ plaque deposition, as well as 1 year old female APP/PS1 mice which exhibited significantly greater Aβ plaque deposition (Fig. 1A) as previously described^41^. To assess BBB permeability, we infused 40 KDa FITC-dextran tracer intravenously. After FITC-dextran injection, the mice were perfused with cold PBS to remove intravascular FITC-dextran, thus allowing for the quantification of the remaining extravasated FITC-dextran that had leaked into the parenchyma. As shown in Fig. 1B, brain vascular leakage is already observed in the frontal cortex of female APP/PS1 mice as early as 6 months. Quantification of the extent of vascular leak showed a marked increase (p-value < 0.05 and power equals 1) with aging of the APP/PS1 mice (Fig. 1C) with a positive correlation between Aβ deposition and FITC-dextran leakage (Fig. 1D, *Pearson correlation coefficient* (r) = 0.7265, p = 0.0006).

**Figure 2:**
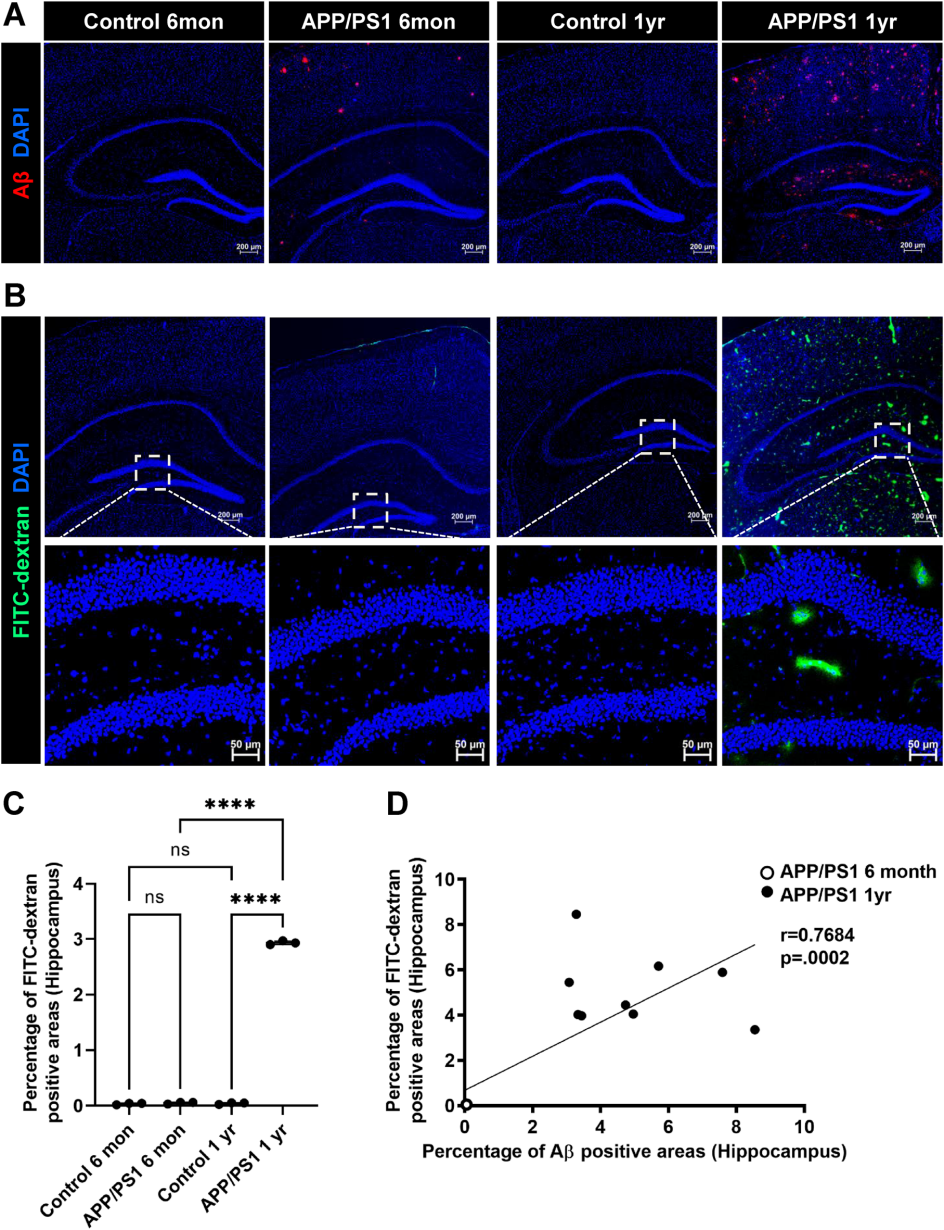
Blood–brain barrier leakiness and Aβ deposition in the hippocampus of APP/PS1 female mice. Control female age-matched and APP/PS1 female mice were analyzed at ages of 6 months and 1 year old for amyloid plaque deposition by staining with antibodies against Aβ (red) and BBB permeability assessed by FITC-dextran leakage (green). Low-magnification representative images of coronal sections of the hippocampus for Aβ (**A**) and of FITC-dextran leak (**B**). The ROI is outlined with a white box and subsequent images are given at higher magnification of tissue corresponding to the ROIs for controls and transgenic mice. Nuclei were counterstained using DAPI. Experiments were repeated at least three times with similar results. Increased BBB leak was observed in the 1 year old APP/PS1 female mice. Scale bars, 200 μm and 50 μm respectively. (**C**) Quantification of FITC-dextran leakage in the hippocampus of the above groups. Data are represented as means and SD for three biological replicates and analyzed by one-way ANOVA followed by Tukey’s multiple comparisons test. ****p = 0.0001, *ns* non-significant. (**D**) Correlation of Aβ deposition and FITC-dextran leakage from 6 months and 1 year old APP/PS1 female mice. Increased FITC-dextran leak was correlated with age-dependent Aβ accumulation. Correlation coefficients and p value are indicated. *r* Pearson’s coefficient, *p* significance.

We next examined the hippocampus, a region which has also been reported to exhibit significant Aβ plaque deposition in AD^41,44^. As shown in Fig. 2A, few Aβ plaques were present in the hippocampus of 6-month-old female APP/PS1 mice, but these levels were markedly increased by the age of 12 months. We observed only minimal vascular leak in the hippocampus in the younger mice (Fig. 2B), and quantification showed a drastic increase (p-value < 0.05) in hippocampus vascular leak by 12 months (Fig. 2C) as well as positive correlation between Aβ deposition and FITC-dextran leakage (Fig. 2D, *Pearson correlation coefficient* (r) = 0.7684, p = 0.0002). These results suggest that BBB permeability is present in the cortex as early as 6 months in the female APP/PS1 model and that BBB leak shows regionality with the cortex showing greater permeability than the hippocampus during the early phases of Aβ deposition in this experimental model of Aβ amyloidosis.

### Transcriptomic analysis of the brain endothelium in APP/PS1 mice

**Figure 3:**
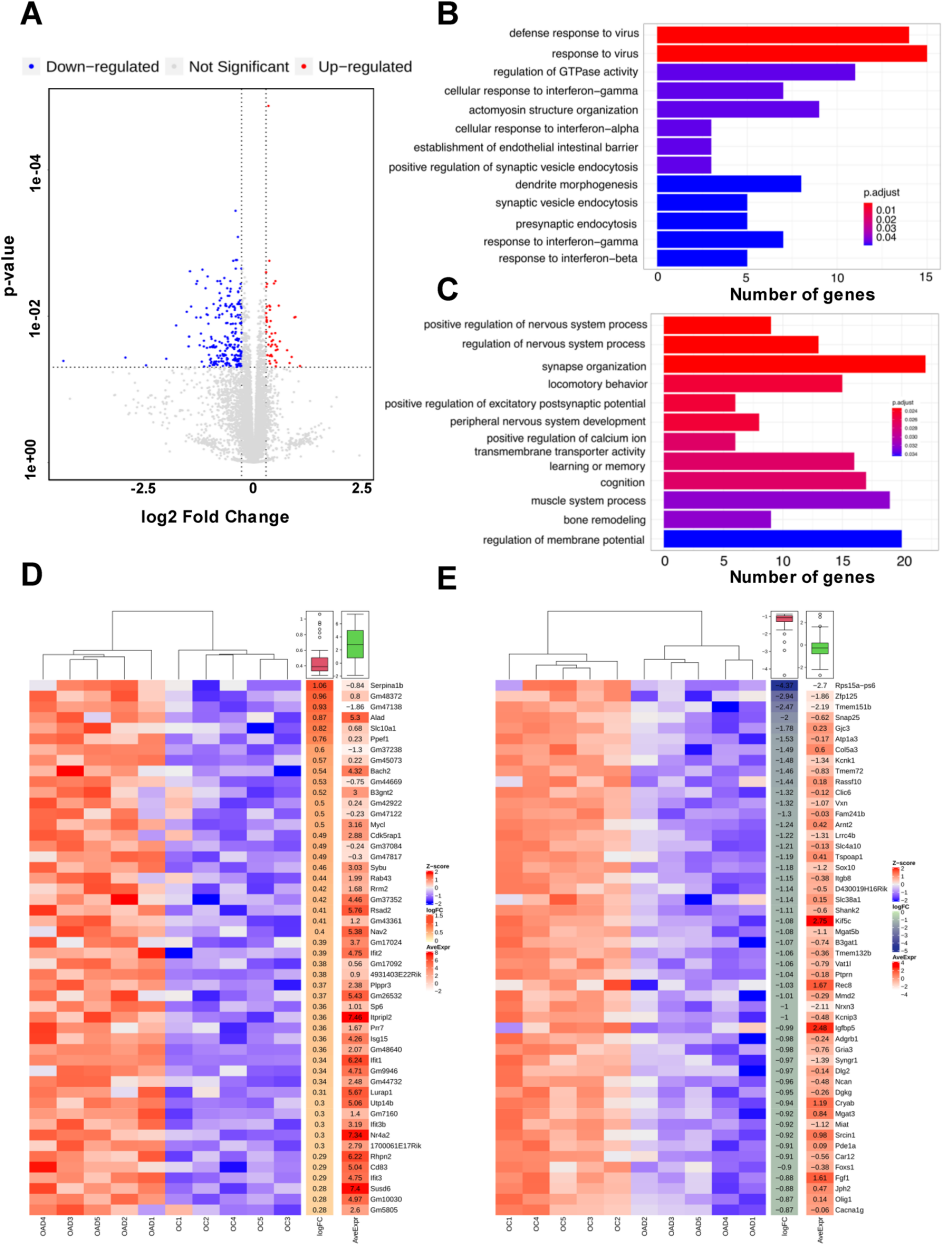
Transcriptomic analysis of brain endothelium in 1 year old APP/PS1 female mice and age-matched control female mice. (**A**) Volcano plot of DE mRNA transcripts analyzed in 1 year old APP/PS1 female mice against age-matched control female mice. The horizontal axis is the log2 fold change of transcripts. The vertical axis is statistically significant. Each dot represents an individual transcript (blue: down-regulated transcript; red: upregulated transcript; grey: no significant difference). The vast majority of differentially expressed genes are downregulated in the APP/PS1 female mice. (**B**) Gene ontology (GO) enrichment analysis of upregulated DE genes in the APP/PS1 female group versus control female group. (**C**) GO enrichment analysis of downregulated DE genes in the APP/PS1 female group versus control female group. Genes were considered upregulated in disease conditions if they were increased log2(fold change) > 1.00 with p < 0.05 and considered downregulated if log2(fold change) < − 1.00 with p < 0.05. The horizontal axis represents the number of genes for each GO term. (**D**, **E**) Heat map of the top 50 differentially expressed (upregulated and downregulated) endothelial genes between APP/PS1 females and control females. DEG (p < 0.05 and |log2 fold change| > 1), and their average expression values are presented in the side columns Each row represents one gene. Orange denotes higher gene expression and blue represents lower expression. Intensity of color is determined by a *Z*-score normalized by gene.

Although brain vascular leak has been associated with AD, less is known about molecular mechanisms that would mediate the increase in endothelial barrier permeability. We therefore performed an unbiased transcriptomic analysis of freshly isolated BECs by bulk RNA sequencing. CD31^+^CD45^−^ BECs were isolated from the cortex of 1 year old female APP/PS1 mice as well as age-matched control female mice using microbeads and evaluated the cell purity by FACS analysis showing that the purity of BECs was approximately 96–99% (Supplementary Fig. S1). To identify the molecular signatures of BECs in the APP/PS1 mice, we evaluated differentially expressed genes (DEGs) using a false discovery rate (FDR) adjusted P-value < 0.05 and at least a two-fold change in expression levels. Our RNA-seq analysis identified 616 DEGs (FDR < 0.05, 220 upregulated and 396 downregulated) out of 14,106 total genes from the control and APP/PS1 groups (Supplementary Fig. S2 and Supplementary dataset). As shown in Fig. 3A, most of the DEGs in BECs comparing female APP/PS1 and age-matched female control mice showed asymmetric regulation with the majority of genes being downregulated.

We next performed gene ontology (GO) analyses on the significant DEGs to assess which biological processes were dysregulated in the BECs isolated from female APP/PS1 mice. The identified biological pathways analyzed by GO enrichment analysis are shown in Fig. 3B and C, and the individual DEGs with the most significant changes are shown in Fig. 3D and E. Importantly, GO analysis revealed upregulation of cellular stress and activation of the innate immune system such as the response to interferons (Fig. 3B) in brain endothelial cells, whereas GO pathway analysis of downregulated DEGs showed downregulation of genes involved in maintenance of neuronal circuitry and transport (Fig. 3C).

**Figure 4:**
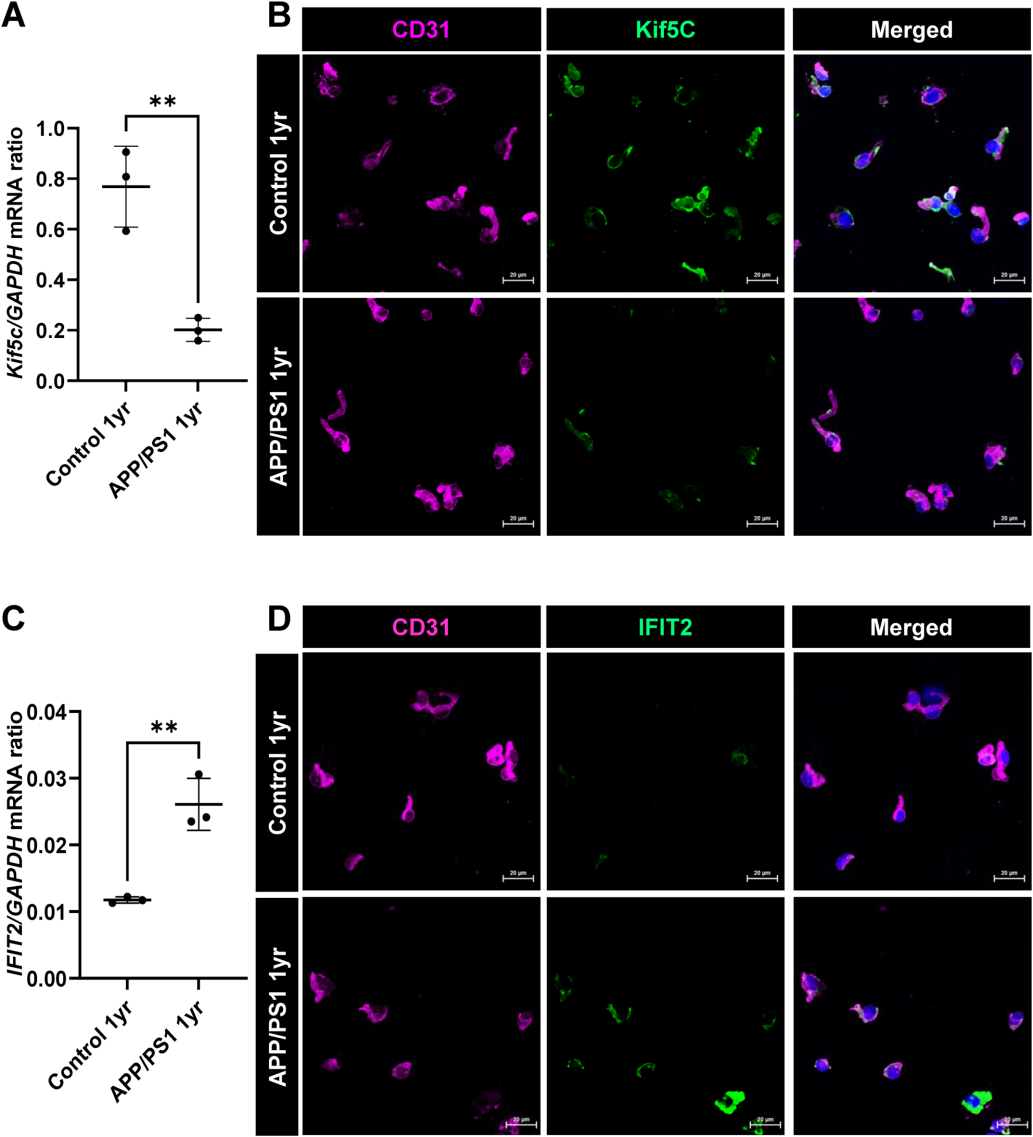
Validation of transcriptomic changes in the brain endothelium of APP/PS1 female mice. qPCR of RNA-Seq data based on log fold change and gene expression levels in brain EC of 1 year old APP/PS1 female and age-matched female control mice. Endothelial cells from the cortex of 1 year old APP/PS1 female mice demonstrated decreased *Kif5c* (**A**) and increased *IFIT2* (**C**) gene expression supporting the RNA-Seq data. Data are represented as means and SD for three biological replicates and analyzed by Student’s T-test. **p = 0.0041; 0.0032 respectively. *GAPDH* is used as a housekeeping gene. (**B**, **D**) Representative confocal images of cytospin of brain EC from cortex of 1 year old APP/PS1 female and age-matched female controls further validated the qPCR results. Scale bar 20 µm.

We then validated our RNA sequencing results in freshly isolated BECs using qPCR and immunocytochemistry. As an example of a downregulated gene in BECs in female APP/PS1 mice, we chose the *Kif5c* gene, that encodes the 5c isoform of the kinesin heavy chain and is critical for cytosolic transport of proteins^45^. qPCR analysis of mRNA obtained from freshly isolated BECs from female APP/PS1 mice confirmed downregulation of this gene compared to age-matched control mice (Fig. 4A). In addition, we performed immunofluorescence on freshly isolated BECs to assess whether protein levels of Kif5c are also decreased. As shown in Fig. 4B, BECs from female APP/PS1 mice demonstrated significantly lower Kif5c staining when compared to BECs from control female mice. Conversely, we also validated the upregulation of the *IFIT2* gene, a member of a family of Type I interferon-induced protein with tetratricopeptide repeats (IFITs)^46,47^. By qPCR analysis, we observed significant upregulation of *IFIT2* from female APP/PS1 mice compared with BECs from age-matched female control mice (Fig. 4C). Furthermore, immunofluorescence of freshly isolated BECs from the cortex of female APP/PS1 mice demonstrated significant elevation in IFIT2 protein levels (Fig. 4D), consistent with an activation of the Type I interferon response in the BECs from APP/PS1 female mice.

### Increased Type I interferon in the brain of APP/PS1 mice

**Figure 5:**
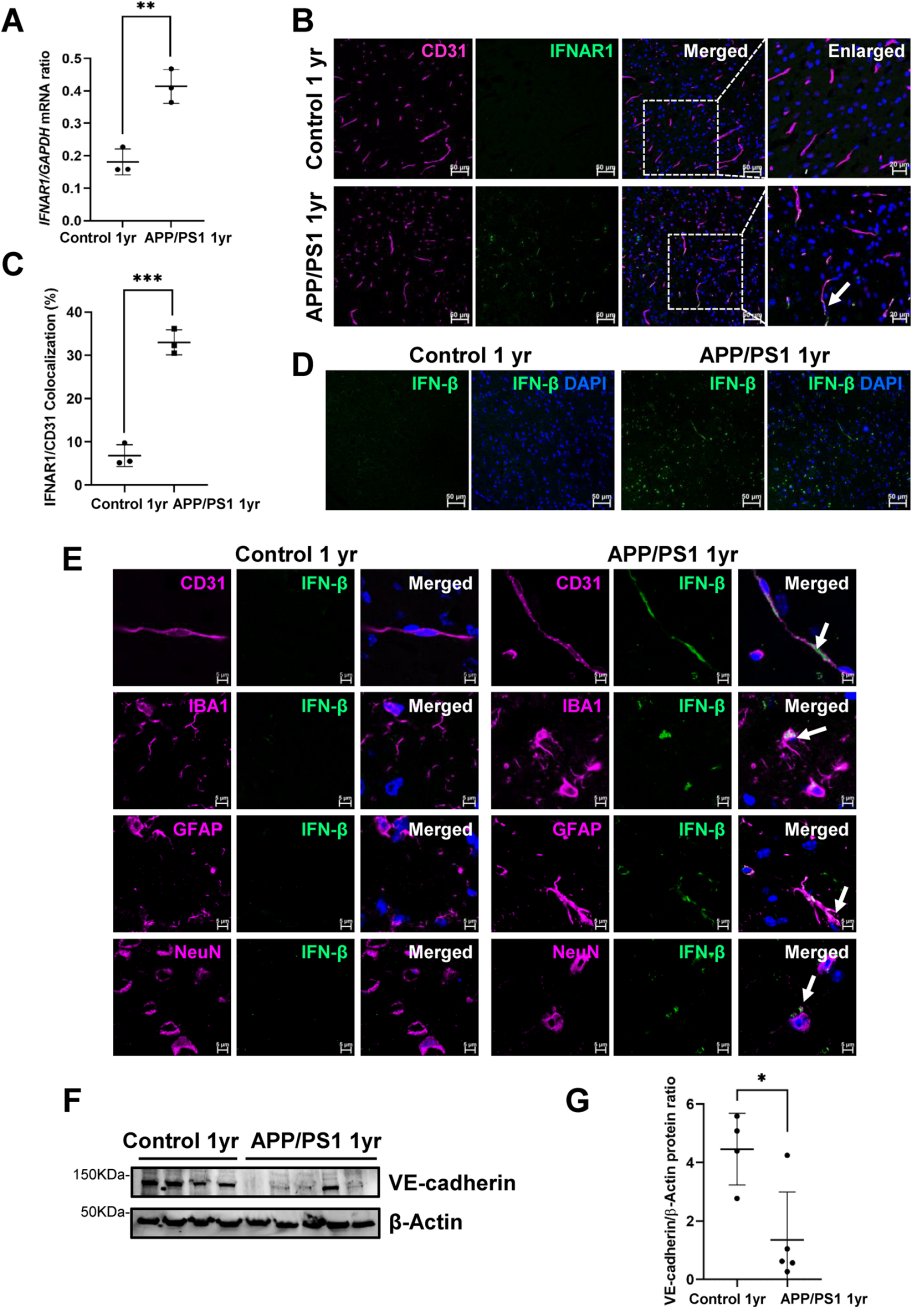
Increased Type I interferon in the brain of APP/PS1 female mice. (**A**) Endothelial cells from cortex of 1 year old APP/PS1 females demonstrated increased *IFNAR1* expression compared to age-matched control females. qPCR data are represented as means and SD for three biological replicates and analyzed by Student’s T-test. **p = 0.0035. *GAPDH* is used as a housekeeping gene. (**B**) Low magnification representative images showing staining patterns for IFNAR1 receptor. Some of the EC (anti-CD31, magenta) from the cortex of 1 year old APP/PS1 female mice showed relatively stronger membrane IFNAR1 (anti-IFNAR, green) staining as compared to age-matched control female mice cortex (enlarged images on right). Nuclei were stained with DAPI. Scale bar: 50 μm and 20 μm respectively. Representative of n = 3 mice per group. (**C**) Quantification of the percentage of EC expressing the IFNAR1 receptor. Percentages are calculated from three sections per mice and averaged. Data are represented as means and SD for three mice for each group and analyzed by Student’s T-test. ***p = 0.0003. (**D**) Low magnification representative images from cortex of 1 year old APP/PS1 female mice showing increased IFN-β expression in 1 year old APP/PS1 female mice compared to age matched female controls. (**E**) Increased expression of IFN-β in endothelial cells (CD31), microglia (IBA), astrocytes (GFAP) and neurons (NeuN) in the cortex of 1 year old APP/PS1 female mice**.** Representative images of immunofluorescent double staining show the co-staining of IFN-β (green) with endothelial cells CD31(magenta), with microglia cells IBA-1 (magenta), with astrocytes GFAP (magenta), and with neurons NeuN (magenta). Arrows indicate colocalization. Nuclei were counterstained using DAPI. Scale bar: 50 μm and 5 μm respectively. (**F**) VE-cadherin protein expression in the cortex of 1 year old APP/PS1 female mice and age-matched female controls. VE-cadherin levels are significantly decreased in the APP/PS1 female mouse brain cortex compared to female controls and the decrease is quantified by NIH image J software. β-Actin is used as loading control and the data is quantified in (**G**). Original blot is presented in Supplementary Fig. S6. Data are represented as means and SD for four control female mice and for five APP/PS1 female mice. Statistical analysis was performed by Student’s t-test. *p = 0.0165.

As Type 1 interferons (IFN-α and IFN-β) transmit its signal via the Type 1 interferon receptor IFNAR1, we next evaluated the expression of IFNAR1 in BECs freshly isolated from the cortex of 1 year old female APP/PS1 and age-matched female control mice. Interestingly, we found that BECs from female APP/PS1 mice showed increased IFNAR1 expression compared with age-matched control female mice (Fig. 5A). As BEC isolation could itself have activated BECs and increased expression of the pro-inflammatory IFNAR1 receptor, we next assessed IFNAR1 expression in vivo and observed elevated levels of this receptor in brain ECs of female APP/PS1 mice compared with age-matched female control mice (Fig. 5B), and in situ colocalization analysis showed a four-fold upregulation of IFNAR1 in BECs of female APP/PS1 mice (Fig. 5C). In light of recent evidence that IFN-β plays a key pathogenic role in AD^48^, we analyzed the expression of IFN-β in the cortex of 1 year old female APP/PS1 mice and compared the expression to age-matched female control mice. We found increased expression of IFN-β in the cortex of 1 year old female APP/PS1 mice (Fig. 5D). Interestingly, IFN-β colocalized with microglia (IBA1), astrocytes (GFAP), endothelial cells (CD31) and neurons (NeuN) (Fig. 5E) indicating that IFN-β production from multiple cell types likely contributes to these higher levels. Of note, IFN-β expression was undetectable in the corresponding brain regions of age-matched female control mice. We next examined whether the release of IFN-β in female APP/PS1 mice could mechanistically affect blood brain barrier (BBB) integrity by investigating the expression of the adherens junction protein VE-cadherin, a key mediator of BBB integrity^38^. A significant decrease in VE-cadherin levels was detected in the cortex of 1 year old female APP/PS1 mice compared to age-matched female control mice (Fig. 5F–G).

### IFN-β activation of brain endothelial cells downregulates adherens junction and tight junction proteins

**Figure 6:**
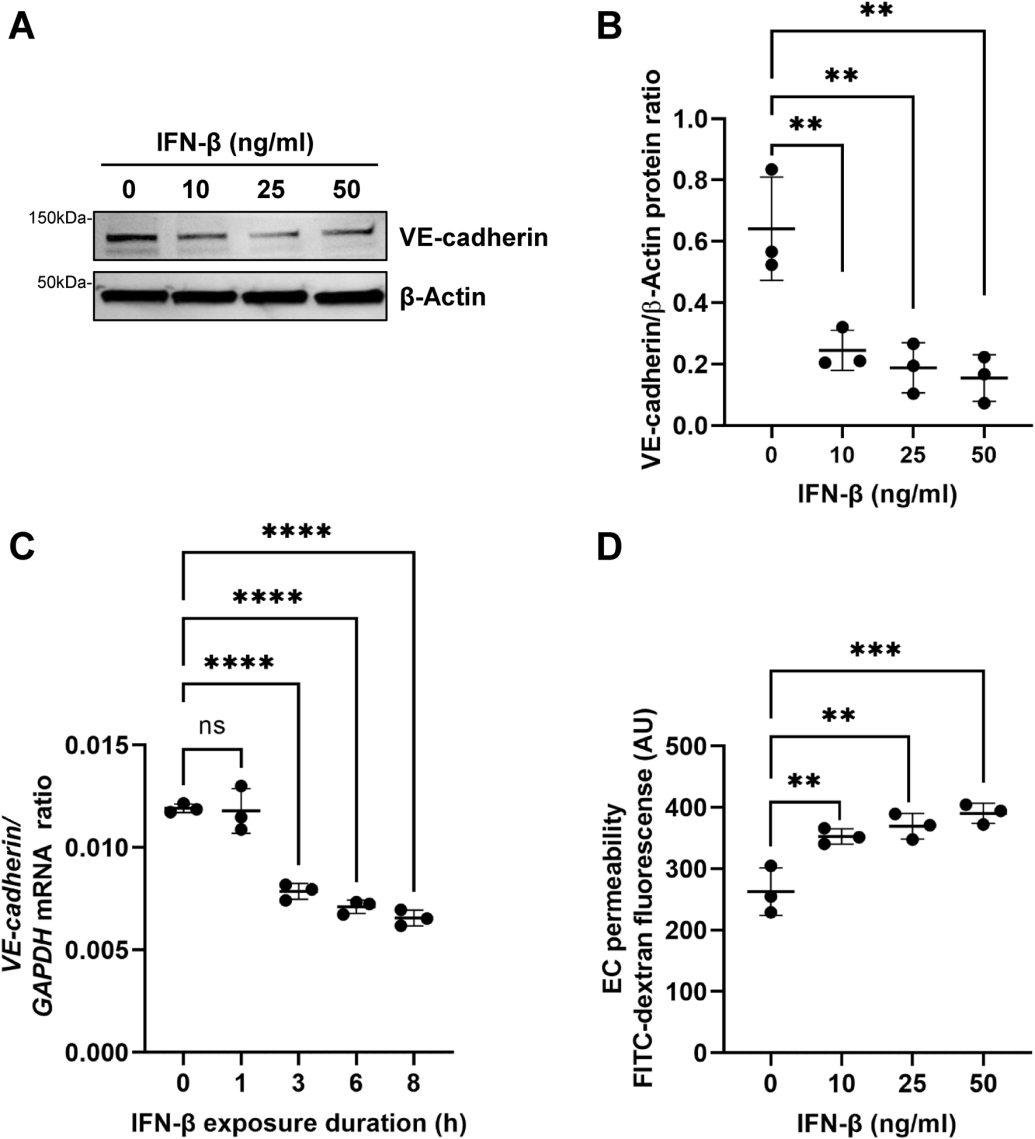
IFN-β downregulates VE-cadherin and increases permeability of human brain endothelial cells. (**A**) Human brain endothelial cells from the hCMEC/D3 cell line were treated with different doses of IFN-β as indicated for 24 h and western blot analysis of total lysates was performed to determine the expression of junction proteins. The immunoblots shown are representative of three independent experiments. VE-cadherin levels are substantially decreased in a dose-dependent manner and the decrease is quantified by NIH image J software. β-Actin is used as a loading control and the data is quantified in (**B**). Original blot is presented in Supplementary Fig. S7. (**C**) VE-cadherin levels were quantified by quantitative RT-PCR in hCMEC/D3 cells after IFN-β (100 ng/ml) stimulation for indicated time points. (**D**) IFN-β treatment dose dependently increased the leak of 40 KDa FITC-dextran through the monolayer of endothelial cells. Data represent the mean ± SD of three independent experiments. Statistical analysis was performed by one-way ANOVA followed by Tukey’s multiple comparisons test. **p = 0.0078, **p = 0.0035, **p = 0.0022; ****p = 0.0001; **p = 0.0082, **p = 0.0029, ***p = 0.0009; respectively. *ns* non-significant.

To mechanistically study the effect of IFN-β on endothelial barrier integrity, human immortalized brain endothelial cells hCMEC/D3 (D3-BECs) were exposed to recombinant human IFN-β for 24 h and analyzed expression of VE-cadherin. As shown by immunoblot analyses, IFN-β decreased brain endothelial VE-cadherin levels in a dose-dependent manner when compared with vehicle treated D3-BECs (Fig. 6A,B). Next, we investigated the expression of the TJ proteins Occludin and Claudin-5 to determine whether the IFN-β effects are specific for AJ or also affects TJ proteins. As observed in Supplementary Fig. S3, IFN-β exposure also decreased the tight junction proteins Occludin and Claudin-5 levels but the decreases were not as prominent as the IFN-β-induced downregulation of VE-cadherin. We did not observe any cytotoxic effect of IFN-β on D3-human brain endothelial cells at that concentration used for treatment (Supplementary Fig. S4) suggesting that IFN-β inhibits expression of VE-cadherin in D3-BECs independent of inducing cell death.

Since VE-cadherin expression and organization at AJs is an important determinant of vascular integrity^49^, we investigated the molecular mechanisms underlying IFN-β-induced decreased VE-cadherin protein levels. We next verified whether decreased VE-cadherin protein levels were due to decreased transcription of VE-cadherin by qPCR. As observed in Fig. 6C, IFN-β significantly decreased *VE-cadherin* mRNA levels relative to controls as early as 3 h following IFN-β exposure. Next, the effects of IFN-β on vascular permeability were examined in a confluent monolayer of D3-BECs cells using 40KDa FITC-dextran tracer. Compared with the control condition, IFN-β exposure significantly increased the leakage of FITC-dextran through the monolayer in a dose-dependent manner (Fig. 6D). To visualize IFN-β-induced junctional disruptions in D3-BECs, the cell monolayer was stained with a cell stain and reduced staining intensity was observed compared to controls (Supplementary Fig. S5A).

### IFN-β induced vascular leak in brain endothelial cells is mediated via the Type I interferon receptor IFNAR1

**Figure 7:**
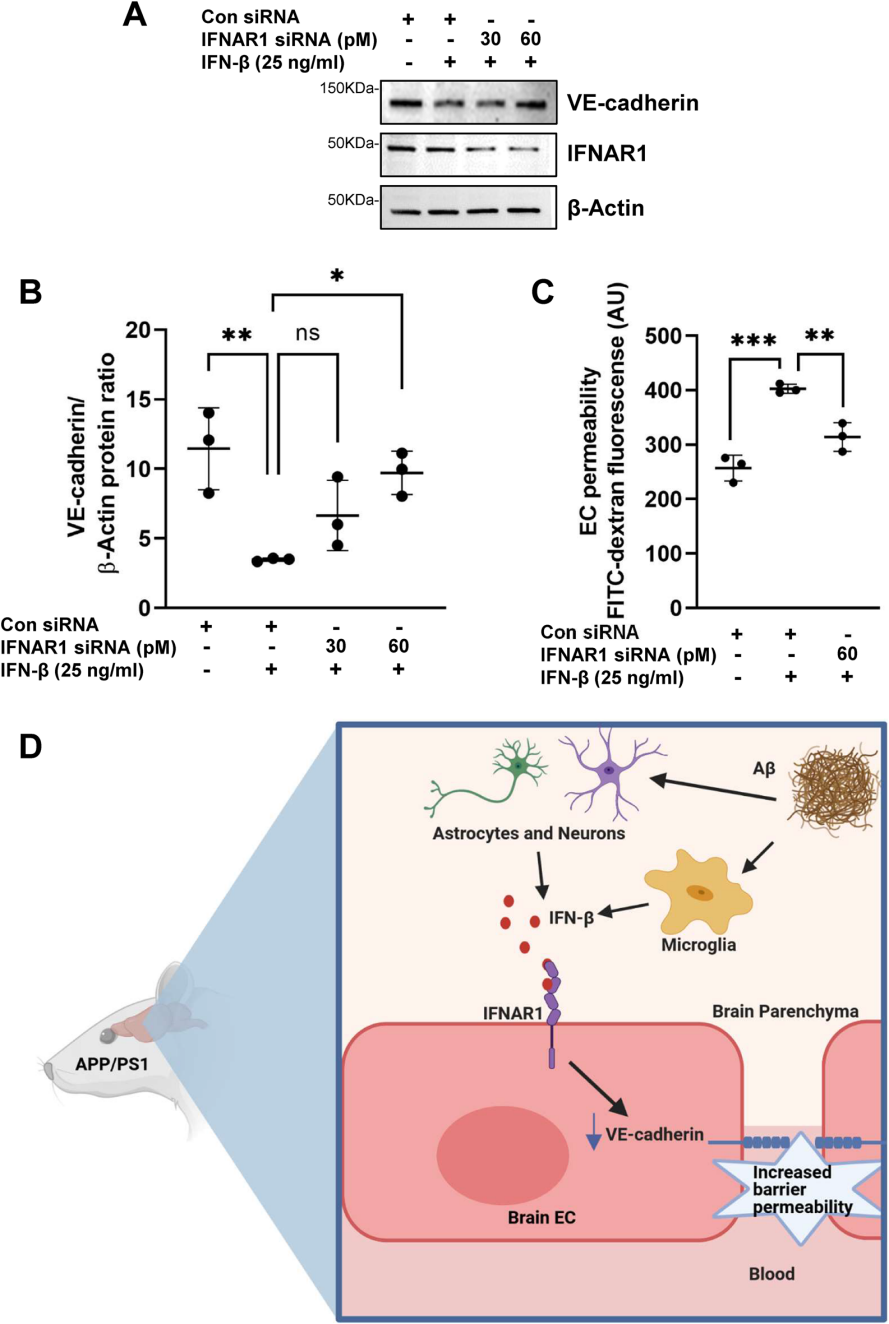
IFN-β induced leakiness in the brain endothelial barrier is mediated via the receptor IFNAR1. (**A**) hCMEC/D3 cells were treated with IFNAR1 siRNA for 48 h and then stimulated with IFN-β for an additional 24 h. IFN-β induced VE-cadherin downregulation was abrogated after transient knockdown of IFNAR1 implicating the involvement of IFN-β signaling through IFNAR1 receptor. β-actin is used as a loading control. Original blot is presented in Supplementary Fig. S8. (**B**). VE-cadherin levels are quantified by image J software. (**C**) IFNAR1 siRNA treatment significantly reversed the IFN-β induced increase of endothelial permeability. Data analyzed by one-way ANOVA followed by Tukey’s multiple comparisons test. *p = 0.0263, **p = 0.0068; **p = 0.0049, ***p = 0.0003 respectively. N = 3 independent experiments. (**D**) Schematic representation of possible mechanisms regulating blood brain barrier breakdown in AD. Aβ-induced release of IFN-β from microglia, astrocytes, endothelial cells and neurons disrupts the BBB integrity via IFNAR1 expressed in brain endothelial cells.

We then investigated whether the induction of VE-cadherin downregulation and the increase in endothelial barrier leak was mediated by the Type I interferon receptor IFNAR1. Depletion of IFNAR1 using siRNA prevented VE-cadherin downregulation whereas the scrambled siRNA control did not affect VE-cadherin levels (Fig. 7A,B). Importantly, depletion of IFNAR1 also abrogated IFN-β induced EC permeability when compared to the scrambled siRNA control (Fig. 7C and Supplementary Fig. S5B). Taken together, these results indicate that the increase in brain endothelial permeability induced by IFN-β is mediated via the Type I interferon receptor IFNAR1 (Fig. 7D).

## Discussion

The present study characterizes the brain endothelial barrier permeability and transcriptome in an experimental model of Alzheimer’s Disease. The major novel findings of the present study include the following: (1) subtle impairment of BBB integrity and limited Aβ deposition was observed in the cortex of 6 month old female APP/PS1 mice, followed by increased deposition of Aβ and severe compromise of BBB integrity in the frontal cortex and hippocampus of 1 year old female APP/PS1 mice; (2) An unbiased transcriptomic analysis showed significant upregulation of IFN-β signaling and significant VE-cadherin downregulation in the brain endothelium of the cortex of 1 year old female APP/PS1 mice as compared to age-matched female controls; (3) IFN-β treatment in vitro decreased the levels of both tight junction (Occludin, Claudin-5) and adherens junction (VE-cadherin) proteins; (4) depletion of the type I interferon receptor IFNAR1 in brain endothelial cells prevented the IFN-β-induced degradation of VE-cadherin as well as the induction of barrier leakiness.

BBB leakiness, as indicated by increased FITC-dextran leakage in the mouse model of AD, was associated with increased levels of Aβ. This is consistent with the finding that Aβ deposition can lead to increased microhemorrhages and infiltration of plasma proteins into the CNS^2^. Furthermore, the APOE ε4 allele, the strongest known genetic risk factor for developing late-onset AD, is associated with a greater incidence of CAA and cerebrovascular alterations^11,50,51^. We found that the BBB leakage at an early age (6 months) was primarily located in the frontal cortex but not in the hippocampus of the female APP/PS1 transgenic mice (Fig. 2B). Of note, the degree of vascular leak in the cortex and hippocampus was positively correlated with the extent of Aβ deposition, suggesting that blood–brain barrier breakdown may follow Aβ accumulation in APP/PS1 mice. Regional heterogeneity of microglia and astrocytes has been observed in the adult human brain, with regards to density, functional markers and transcriptomic profiles^50,52,53^. A subset of microglia with activated type 1 IFN are found in the hippocampus during the later stages of neurodegeneration which are thought to increase BBB permeability in the hippocampus of the aged AD mouse model^54^.

As AD is a slow progressing neurodegenerative disease, we performed our unbiased transcriptomic analysis of the brain endothelium in 1 year old female APP/PS1 mice as the increased Aβ deposition by this age would likely induce gene expression changes in the brain endothelium. Our transcriptomic analysis identified that the most prominent upregulated gene expression pathways in the brain endothelium of female APP/PS1 mice were “defense response to virus” pathways which primarily indicated activation of interferon signaling as evidenced by interferon signature genes such as the *Ifit* (Interferon-induced protein with tetratricopeptide repeats) family genes *Ifit1, Ifit2 and Ifit3b.* On the other hand, the brain endothelial gene expression pathways that were most downregulated included those involved in the regulation of nervous system processes, neurotransmitter transport or synapse organization such as *Kif5c*, which is a key cytosolic transport and trafficking regulator or myelination related genes including *Ptn*^55^ and *Olig1*^56^. The presence and downregulation of such genes in the endothelium, even though these genes are typically thought to be primarily neuronal, may be surprising. The FACS analysis indicated that the endothelial isolation showed high levels of purity and allowed us to exclude doublets in which neurons would have been adherent to endothelial cells. This observation of “neuronal” genes in the brain endothelial cells is consistent with a recent report that isolation of brain endothelial ribosomes using the Ribotag method as well as single cell RNA-sequencing of the brain endothelium which showed that the brain endothelium expresses genes involved in synaptic vesicle transport as well as other neurotransmitter trafficking related genes^57^. The observation that brain endothelial cells express “neuronal” genes might be due to crosstalk between neurons and endothelial cells via the endothelial uptake of neuronal exosomes, but it could also reflect that the brain endothelium may be responding to environmental cues such as the extracellular matrix and paracrine factors which may program the endothelium to take on a parenchymal signature, similar to what has been observed for the cardiac endothelium which expresses “cardiomyocyte” genes^58^. Our discovery that the brain endothelium downregulates these genes with the progression of amyloid plaque deposition and aging in the APP/PS1 mouse model also raises important questions about whether the expression of such “neuronal” genes in the brain endothelium may contribute to the homeostatic function of neurons.

A recent study identified that Aβ forms complexes with nucleic acids which in turn elicits type I IFN production in microglia to drive neuroinflammation and neurodegeneration in several mouse models of AD^48^. We found that not only microglia but other brain cell types such as astrocytes, neurons and endothelial cells co-localized with IFN-β and that multiple cell types may contribute to increased type I IFN levels observed in the cortex of 1 year old female APP/PS1 mice. Previous studies have suggested that the Type I Interferon IFN-β can induce BBB dysfunction in a stroke model^59^. Our in vivo study showed decreased VE-cadherin levels in the cortex of female APP/PS1 mice compared to age-matched female control mice. In addition, our in vitro studies using human brain ECs showed that IFN-β can directly act on brain endothelial cells and increase EC permeability by downregulating both the AJ protein VE-cadherin as well as the TJ proteins Occludin and Claudin-5 with the downregulation of VE-cadherin being the most prominent. We observed reduced VE-cadherin mRNA levels following IFN-β exposure of brain endothelial cells, but we cannot rule out that IFN-β may additionally induce post-translational downregulation of VE-cadherin and thus promote endothelial barrier integrity. As adherens junctions also stabilize tight junction organization, it is possible that decreased VE-cadherin levels may be an initiating event which could also result in destabilization of tight junctions^60^.

Interferon signaling can function as a double-edged sword in the brain with Type I IFN /Type II IFN balance, thus contributing to distinct brain pathologies^61,62^. Blockade of the interferon signaling in the choroid plexus of aged mice and in the microglia of the AD mouse model decreased inflammation, improved cognitive function and increased hippocampal neurogenesis levels through decreased microgliosis and astrogliosis as well as increased expression of BDNF and IGF, all of which were dependent on type II IFN signaling^62,63^. In contrast, augmentation of type I IFN (IFN-β) signaling in inflammatory CNS diseases such as multiple sclerosis blocked excess type II IFN (IFN-γ) signaling, CD4 T regulatory cell suppression and leukocyte infiltration^62,64,65^. However, there have been cases of IFN-β treated MS patients developing impaired motor functions and showing cognitive deficits. Therefore, IFN-β-therapy may attenuate IFNγ-dependent autoimmune disease^65^, but blockade of IFN-β signaling also restores IFNγ-dependent activity required for proper brain functioning and repair such as in aging and in AD^63^. We would further like to clarify the opposing phenotypes observed in EC barrier function with IFN-β treatment in vitro. It is important to note that previous reports suggest IFN-β can also stabilize the brain endothelial barrier^66–68^ and are thus different from what we observed. These discrepancies could be due to the role of additional mediators released by IFN-β which could additionally regulate the barrier integrity and may have been different in the distinct in vitro experimental settings. Importantly, our in vitro findings on IFN-β induced downregulation of endothelial junction proteins and reduction of barrier integrity match the in vivo observation of increased IFN-β in the AD mouse model being correlated with increased brain endothelial barrier permeability. However, to establish a definitive causal link between IFN-β and brain endothelial barrier integrity in the AD model, it would be necessary to perform genetic studies with endothelial specific deletion of the Type I interferon receptor in vivo, and then assess whether this restores the brain endothelial barrier integrity in the AD mouse model. Although our studies used female mice due to the increased Aβ burden and pathology that has been observed in female APP/PS1 mice^42,43^, we suspect that a similar association between enhanced interferon signaling and increased blood brain barrier permeability is also present in male APP/PS1 mice but it may be interesting to address in future studies whether there is sexual dimorphism in the link between interferon signaling and blood brain barrier endothelial dysfunction.

In summary, we performed unbiased transcriptomic analysis of the brain endothelium and identified increased Type I interferon signaling in the brain endothelium as a putative pathogenic pathway that might be responsible for loss of brain endothelial integrity during the progression of disease. Although our in vitro findings from human immortalized brain endothelial cells do not truly resemble the in vivo context of brain endothelial cells in the brain of AD patients, our results suggest that specific targeting of IFNAR1 may be a potential therapeutic avenue to reduce brain endothelial permeability in Alzheimer’s disease.

## Methods

### Mice

All experimental procedures were conducted in accordance with the protocols approved by UIC Animal Care and use committee (ACC protocol 18-248) and following the National Institutes of Health guidelines. Female APP/PS1Swe/PSEN1DE9 (known as APP/PS1 in this study) mice were housed in the UIC animal facility in temperature, humidity and light controlled rooms with free access to food and water in accordance with the guidelines for the care and use of lab animals adopted by UIC. We used female 6 month and 1 year old APP/PS1 mice and age matched female controls with the same genetic background (C3H/BL6). Genotyping of the mice was performed by PCR analysis of tail DNA. The status of in-house-mice were monitored every 2–3 months via weight and physical checks; young 6-month-old mice weighed around 30 g while mice around 1 year old weighed 40–50 g. Mice found to have health issues as assessed by the in house veterinary medical officer were excluded from studies. Mice were anaesthetized and transcardially perfused before removal of brain tissue. This study is reported in accordance with ARRIVE guidelines.

### Preparation of endothelial cells from mouse adult brain

We followed a dissociation protocol optimized for isolation of single brain vascular cells with some modifications^69^. Briefly, 1 year old APP/PS1 female mice as well as age-matched female control mice were perfused with ice cold PBS. Following perfusion, the meninges were removed and the cortex was microdissected on ice and gently minced into smaller pieces using a sterile chilled razor blade and resuspended in 2.5 ml HBSS without calcium and magnesium containing collagenase/dispase and DNase (Worthington Biochemical LK003178). The resuspended cortex was incubated for 60 min at 37 °C. Halfway through the incubation the cell suspension was triturated three times using Pasteur pipettes. The cell suspensions were then resuspended in 2.5 ml ice-cold HBSS, 2 mM EDTA and 0.5% BSA and the cells were pelleted at 300×*g* at 4 °C for 5 min. The cell suspension was then passed through a prewetted 40 μM cell strainer into a prechilled 50 ml conical tube and centrifuged at 300×*g* at 4 °C for 5 min. Myelin removal beads (Miltenyl Biotec, 130-096-433) and LS columns (Miltenyl Biotec, 130-042-401) were used according to the manufacturer’s protocol to remove myelin debris. The resulting flow-through after myelin removal contains single cells suspension of microglia, astrocytes and endothelial cells. After myelin removal, CD31 cells were further enriched by using CD31 microbeads (Miltenyl Biotec, 130-097-418). The purity of isolated endothelial cells was about 97% (CD31 positive and CD45 negative) as assessed by flow cytometry (Supplementary Fig. S1).

### Flow cytometric analysis of brain EC purity

Part of isolated brain ECs were blocked with anti-Fc CD16/32 (1:100, BD Biosciences) before immunostaining with fluorescently labelled antibody cocktail specific for leukocyte/hematopoietic lineage marker CD45 (PE-conjugated; 1:100; eBiosciences) and the endothelial surface marker CD31 (APC-conjugated, 1:100, eBiosciences) or isotype control antibodies (APC and PE-conjugated, 1:100, eBiosciences). Cells were then analyzed by flow cytometry on the Beckman Coulter Cyan ADP Flow cytometry analyzer and Summit 4.3 software. Cell debris and dead cells were excluded from the analysis based on scatter signals.

### Preparation of cytospin slides from brain EC cell suspension

The cytospin slides for EC were prepared as described previously^57^. Briefly, the frosted upper part of the slide along with the two-holed filter card was inserted into the slideclip (holder) and the cytofunnel was positioned properly so that the holes match up. The required volume of cell suspension was gently pipetted into the cytofunnel after cell counting and calculation and the slide was centrifuged at 500 rpm for 5 min using Thermo Shandon cytospin. After 10 min of 4% PFA fixation the slides were stored in 1 × PBS at 4 °C for immunofluorescence studies.

### Blood brain barrier permeability assay in vivo

Mice were injected with 0.1 ml of 40 KDa FITC-conjugated dextran (Sigma-FD40S) 4 mg/100 μl into the tail vein (n = 3 mice per group). After 15 min of administration, mice were transcardially perfused with ice-cold PBS for 5 min at 12 rpm (4 ml/min) to get rid of the intravenous FITC-dextran. Microscopic visualization of FITC-dextran was done on brain cryosections (10 μM) using Zeiss LSM 880 Confocal Laser Scanning Microscope.

### Quantification of blood brain barrier permeability

Tissue sections labelled with FITC-dextran for detection of FITC-dextran that leaked into the parenchyma were imaged (n = 3 mice per group). The fluorescence of FITC-dextran was measured using Image J and Max entropy was used to quantify the images. The extent of fluorescent extravasation indicating BBB disruption was calculated based on the percentage of the actual area covered by the leakage. For each mouse tissue section, 3 images were acquired within the region of interest (ROI).

### RNA isolation and cDNA synthesis

RNA was extracted from the endothelial cells using TRIzol reagent as per manufacturer’s guidelines. Concentration and purity were assessed using NanoDrop 1000 spectrophotometer (Thermoscientific). 500 ng of RNA was reverse transcribed into cDNA using high-capacity RNA to cDNA reverse transcription kit (Applied Biosystems). The resultant cDNA was diluted 1:2 in RNase-free H_2_O for use in RT-qPCR. Samples were stored at − 80 °C until further use.

### Real time quantitative polymerase chain reaction (RT-qPCR)

RT-qPCR was performed in triplicate in standard 384-well plates using the applied Biosystem QuantStudio 7 Flex Real-Time PCR detection system. The expression of specific mRNAs (*IFIT2*, *Kif5c* and endogenous control *GAPDH*) was assayed using FAST-SYBR PCR Master mix (Applied Biosystems) under the following cycle conditions, 50 °C for 2 min, 95 °C for 20 s, (95 °C for 1 s, 60 °C for 20 s) × 40 repeats. The qPCR mouse primer sequences used were: *Kif5c* Forward-5′-AGAAGTGGAGGACAAGACCAGG-3′; Reverse 5′ GGTGGTTACTAAGCTCTTGCAGC-3′. *Ifit2* Forward 5′-AGTTCTGGCCTTCTGCAGTT-3′; Reverse 5′-GTGTCAAAGCGCTCAAAGCA-3′. *IFNAR1* Forward 5′-AGCCACGGAGAGTCAATGG-3′; Reverse 5′-GCTCTGACACGAAACTGTGTTTT-3′; GAPDH Forward 5′-GGT GAA GGT CGG TGT GAA CG-3′, Reverse 5′-TTG GCT CCA CCC TTC AAG TG-3′. The qPCR human primer sequences used were: *CDH5* Forward 5′-ATGAGATCGTGGTGGAAGCG-3′; Reverse 5′-TGTGTACTTGGTCTGGGTGA-3′. *GAPDH* Forward 5′-CTGATTTGGTCGTATTGGGC-3′; Reverse 5′ TGGAAGATGGTGATGGGATT-3′. The Quant studio Software v1.7.1 was used to generate threshold cycle (Ct) values, and mRNA expression calculated using the ΔCt method.

### Immunohistochemistry

Mice were anaesthetized and transcardially perfused with cold PBS before removal of brain tissue. Brain tissues that were not perfused properly were not used for immunostaining analyses since autofluorescence associated with blood contamination interferes with the analyses. The mice brains were fixed in 4% PFA overnight dehydrated in 30% sucrose solution sectioned at 10 μm with a cryostat. Parallel coronal cryostat brain sections from wildtype and APP/PS1 mice were prepared for immunohistochemistry. Briefly, brain sections were washed three times with PBS containing 0.02% Triton-X 100 and blocked with 10% normal donkey serum blocking buffer in PBS containing 0.02% Triton-X 100 for 1 h at room temperature. Sections were incubated overnight at 4 °C with the following primary antibodies: rabbit polyclonal anti-Aβ antibody for plaques (1:100, ab2539, Abcam), rat monoclonal anti-CD31 antibody (1:20, #550274, BD Biosciences) for blood vessels, mouse monoclonal anti-IBA1 antibody (1:150, NB 1001028, Novus Biologicals) for microglia, NeuN (1:150, MAB 377, Millipore), GFAP (1:150, Santacruz), anti-IFN-β (1:100, NBP177288, Novus Biologicals), anti-IFNAR1 (1:150, NBP2 75534, Novus Biologicals). After three 10 min washes in PBS, sections were incubated for 1 h at room temperature with a corresponding secondary antibody. Secondary antibodies used included the following: Alexa fluor − 488, − 594, or − 647 conjugated secondary antibodies to rabbit, mouse, rat and goat (1:250, all raised in donkey from Jackson Immunoresearch). DAPI is used for nuclear stain. For negative controls, sections were incubated without primary antibody. Sections were rinsed with PBS and sealed with Prolong Gold (Invitrogen) and glass covers. Images were acquired using Zeiss LSM 880 Confocal Laser Scanning Microscope Quantitative image analysis of the Aβ, immunoreactive cortical and hippocampal regions were performed on five fields corresponding to FITC-dextran leakage area and three cortical sections from three mice brain samples. NIH ImageJ software was used for analysis.

### Western blot

Cell lysates from D3 cells and mouse brain cortical lysates were lysed with lysis buffer containing 1 × protease and phosphatase cocktail inhibitor (Roche, USA) and prepared for western blot analysis. Blots were probed with VE-Cadherin (1:1000, R&D), and IFNAR1 (1:1000, Thermo Fisher), Occludin (1:1000, Thermo Fisher), and Claudin-5 (1:1000, Thermo Fisher) exposed to HRP-conjugated secondary antibodies and imaged using enhanced chemiluminescence to detect immobilized antibodies. Densitometric analysis of immunoblots for respective proteins (VE-Cadherin and IFNAR1) was done by using ImageJ software. Digital images were captured by iBright imaging system (Thermofisher Scientific). All the experiments were repeated at least three times under the same conditions.

### Endothelial permeability assay

Briefly, hCMEC/D3 cells were plated into the collagen coated inserts at the density of 0.2 × 10^6^ cells/insert and grown to confluency for 24 h. Then they were treated with different doses of IFN-β for 24 h. After 24 h, 40KDa FITC-Dextran was added to the insert and incubated at room temperature for 20 min for permeation. The mean fluorescence of FITC-Dextran that passed from insert to the receiver well was quantified using a fluorescence plate reader with 485 nm excitation and 535 nm emission. After completion of the permeability analysis by FITC-dextran, the endothelial monolayer was stained with cell stain following manufacturer’s instruction (Millipore In Vitro Vascular Permeability Assay # ECM644) and brightfield imaging of the monolayer was acquired by Olympus IX70 microscope.

### Transcriptomic data preprocessing

The sequenced reads from all replicates and samples were aligned to the mouse (mm10) reference genome with STAR^70^. The aligned reads were quantified to mRNA count table using HTSeq-count^71^. Gene symbols were annotated to the ENSEMBL features using biomaRt package^72–76^. Low count genes were filtered out using filterByExpr function from edgeR package. The count tables were normalized by taking log counts per million using ‘cpm’ function available in edgeR package (Supplementary Fig. S2). The batch correction was performed using ‘ComBat’ function available in ‘sva’ Bioconductor package. To visualize the effects of batch corrections principal component analysis (PCA) and density plots of expression values of each replicate were generated (Supplementary Fig. S2).

### Differential expression analysis

Differential expression analyses between groups 1 year old AD vs 1 year old controls and 6-month-old AD vs young controls were carried out using the limma Bioconductor package. Linear models for each gene were fitted using lmFit function. For fitted linear models moderated t-statistics, moderated F-statistic, and log-odds of differential expression were computed by using empirical Bayes moderation (limma–eBayes). The p values were adjusted to multiple tests with Benjamini–Hochberg method (Fig. 3). A threshold was set for the adjusted p value (< 0.05) to filter the significant genes. To visualize the top differentially expressed genes (DEGs) expression levels across the comparison groups, a normalized gene expression heatmap with corresponding log2FC and average expression was generated.

### GO enrichment analysis

For each comparison, we performed gene ontology (GO) enrichment analysis for both upregulated DE genes and downregulated DE genes. We applied ‘enrich GO’ function provided in cluster Profiler package. The enriched GO categories were recorded in an Excel sheet after FDR control. The gene counts of top categories were visualized in bar plots.

### Statistical analysis

All data are expressed as means ± SD from at least three independent experiments. Statistical analyses included one-way ANOVA with corrections for multiple comparisons, Student t test for comparisons between two groups and Pearson r correlation coefficient analysis. All statistical analysis was carried out using GraphPad Prism software with p < 0.05 considered statistically significant for all experiments. Power analysis was conducted using a balanced one-way ANOVA power analysis function power.anova.test() in R. We calculated the within-group variance and between-group variance and set a significant level to 0.05.

## Supplementary Information

Supplementary Information 1.

Supplementary Information 2.

## Data Availability

The bulk RNA-seq datasets generated and analyzed during this current study are available in the Gene Expression Omnibus (GEO) database with Accession Number GSE206629 (data link, https://www.ncbi.nlm.nih.gov/geo/query/acc.cgi?acc=GSE206629).
