# Supplementary material for: Increased Type I interferon signaling and brain endothelial barrier dysfunction in an experimental model of Alzheimer’s disease
Source: Sci Rep. 2022 Oct 1;12:16488. doi: 10.1038/s41598-022-20889-y (PMC9526723; doi:10.1038/s41598-022-20889-y)
Supplement: Supplementary file 1 — Supplementary Information 1. [file 41598_2022_20889_MOESM1_ESM.pdf]

Supplementary Information

Increased Type I Interferon Signaling and Brain Endothelial Barrier Dysfunction in an Experimental Model of Alzheimer's Disease

Arundhati Jana, Xinge Wang, Joseph W. Leasure, Lissette Magana, Li Wang, Young-Mee Kim, Hemraj Dodiya, Peter T. Toth, Sangram S. Sisodia, Jalees Rehman.

Supplementary Section Inventory:

Supplementary Figures S1-2 relates to main Figure 3

Supplementary Figures S3-4 relates to main Figure 6

Supplementary Figure S5 relates to main Figure 6 and 7

Supplementary Figures S6-9 relates to main Figure 5F, 6A, 7A and supplementary figure S4A

Supplementary data analysis relates to main Figures 1-2

Supplementary dataset. List of the RNA-seq DEGs (separate file)

Supplementary Figures

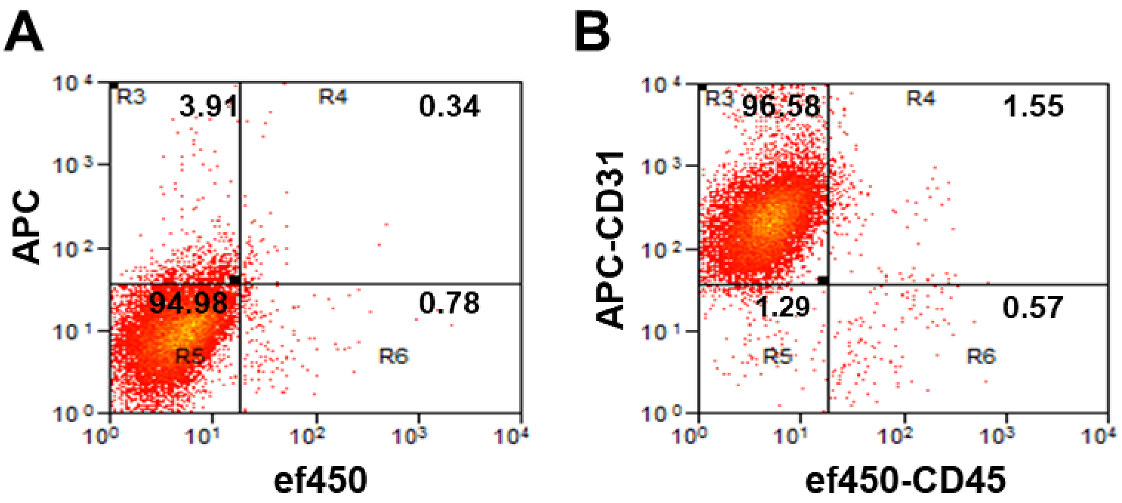

Supplementary Figure S1. Purity of isolated brain endothelial cells. Purity of isolated BEC was analyzed by Flow cytometry using specific antibodies against CD31 and CD45. **A**, a dot plot for negative control cells by staining the isotype antibodies for APC-isotype and ef450-isotype antibodies. **B**, a dot plot for positive cells by staining the APC-CD31antibody for endothelial cells and ef450-CD45 antibody for lymphoid cells. The sorted CD31+ and CD45- cells (**R3** region at **B**) were defined as BEC and the purity of this group was 96.58%.

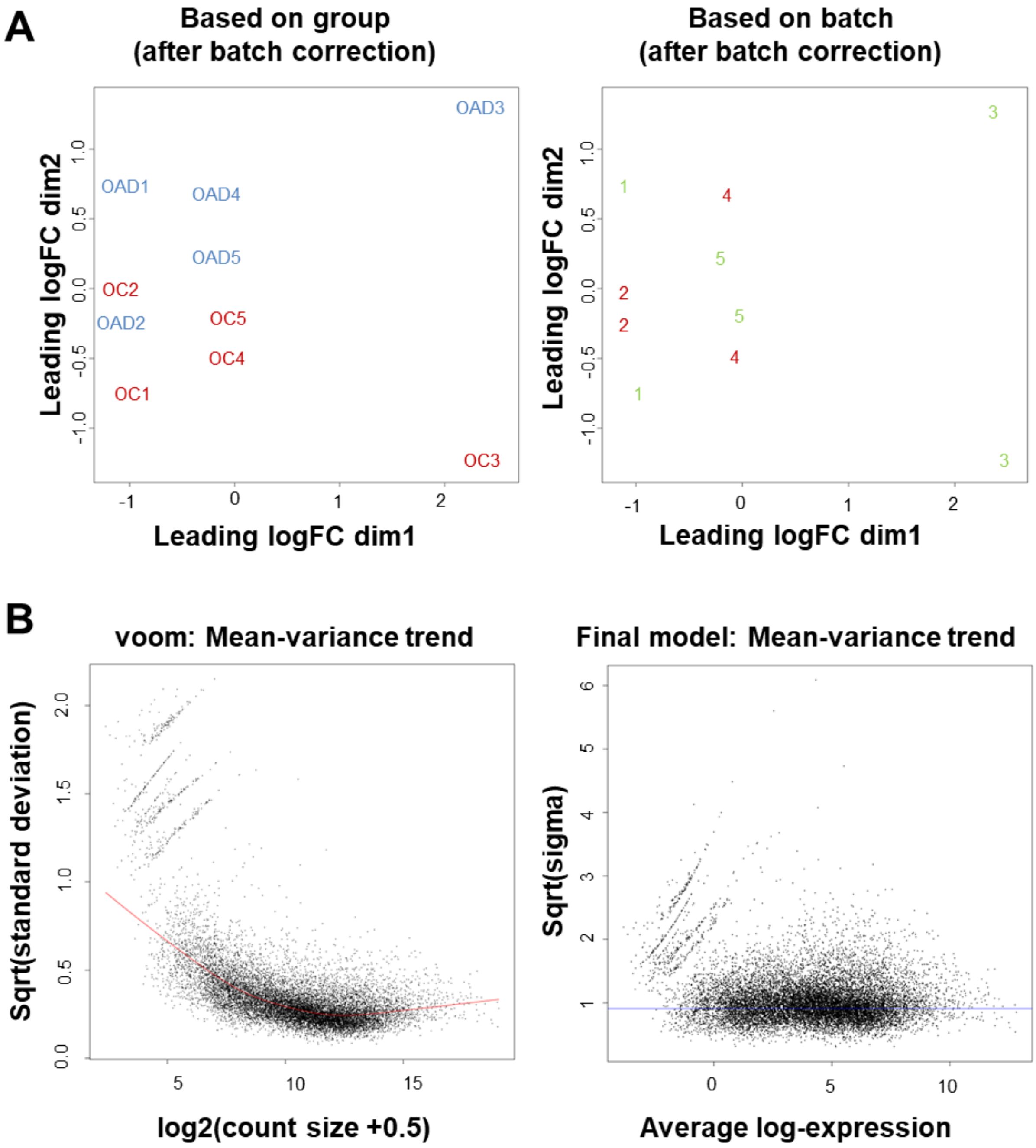

Supplementary Figure S2. Multidimensional scaling plots of two groups. After batch correction, colored by group and batch number of the replicates. Mean-variance trend plots showing before and after data normalization. 1 yr old control female mice (**OC**), 1 yr old APP/PS1 female mice (**OAD**).

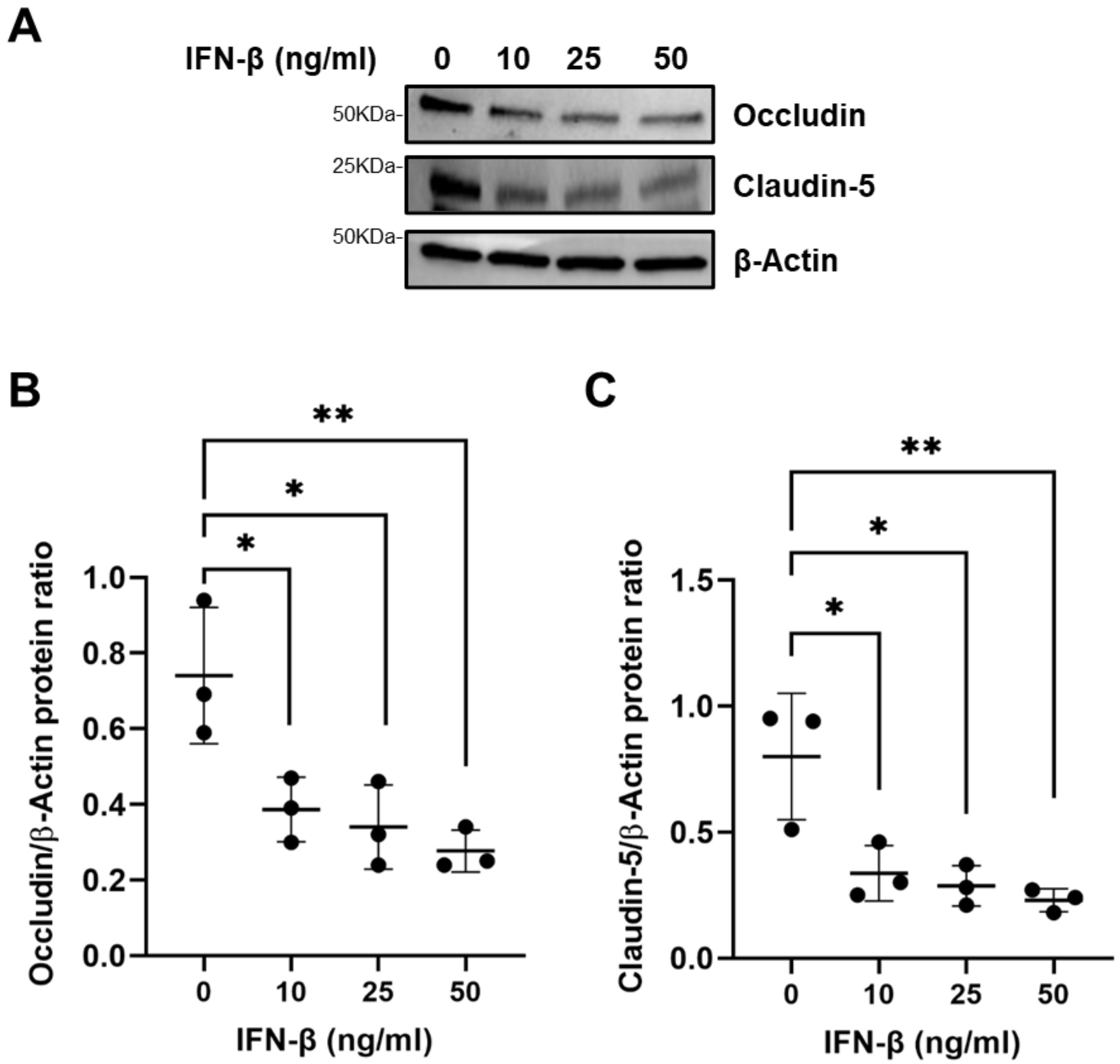

Supplementary Figure S3. IFN-β treatment significantly reduced the levels of the tight junction proteins Occludin and Claudin-5 in human brain endothelial cells. (**A**) Occludin and Claudin-5 expression by immunoblotting in hCMEC/D3 cells treated with increasing doses of recombinant IFN-β for 24h. The immunoblots are representative of three independent experiments. (**B**) Occludin and (**C**) Caudin-5 densitometric analysis was done by Image J software (NIH). β-Actin is used as loading control. Statistical analysis was performed by one-way ANOVA followed by Tukey's multiple comparisons test. *p=0.0254, *p=0.0132, **p=0.0056; *p=0.0185, *p=0.0105, **p=0.0057 respectively.

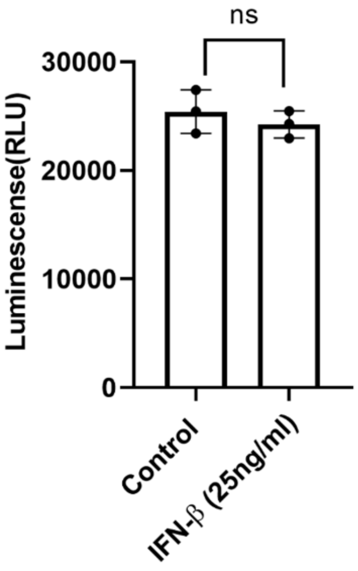

Supplementary Figure S4. Effect of IFN-β on cell viability of human brain endothelial cells. Cell Titer-Glo assay was performed to measure cell viability of hCMEC/D3 cells upon treatment with IFN-β (25 ng/ml) for 24h. RLU, relative light units. ns: non-significant.

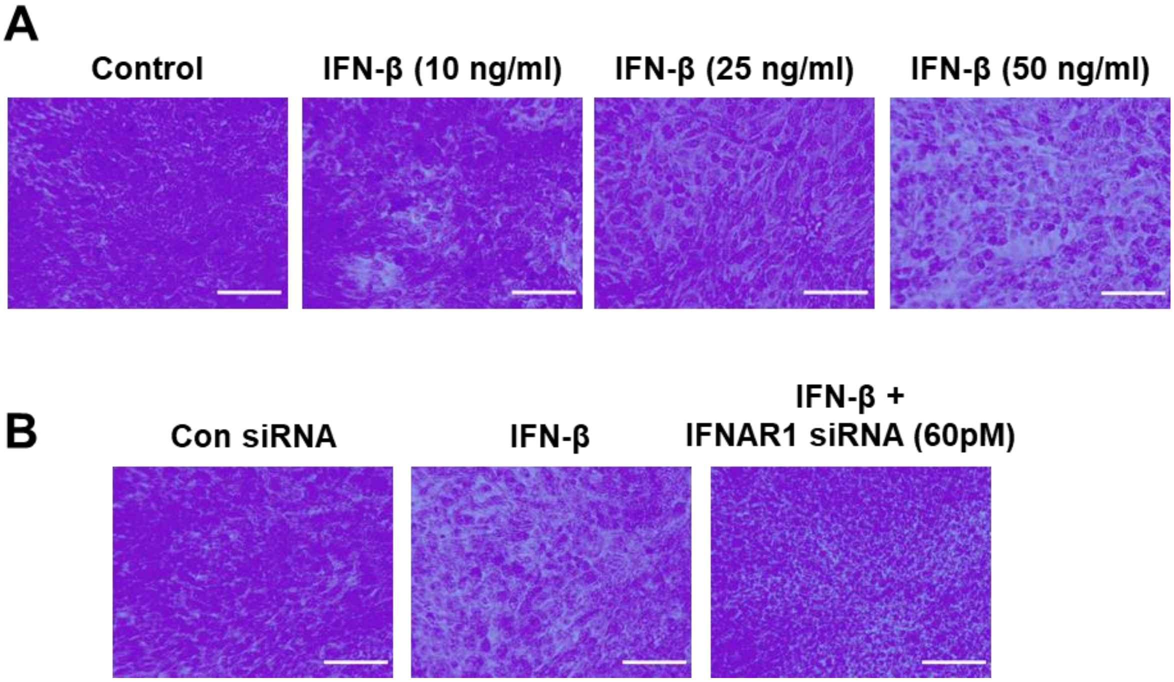

Supplementary Figure S5. IFN-β induced leakiness in the brain endothelial cells is mediated via the receptor IFNAR1. A-B. Brightfield-images of cells stained with cell stain were acquired on an inverted microscope at 20x objective magnification. After completion of the permeability testing by FITC-Dextran, the endothelial monolayer was stained with a cell stain following the manufacturer's protocol (Millipore In Vitro Vascular Permeability Assay # ECM644). The higher color intensity of the monolayer indicates barrier integrity as seen in control cells. In contrast, color intensity diminishes with IFN-β treatment indicating gaps in the monolayer. As observed above, disruption of BEC monolayer integrity was seen following IFN-β treatment (**A**). Interestingly siRNA IFNAR1 significantly blocked IFNβ-induced barrier disruption (**B**). Scale bar 50 µm.

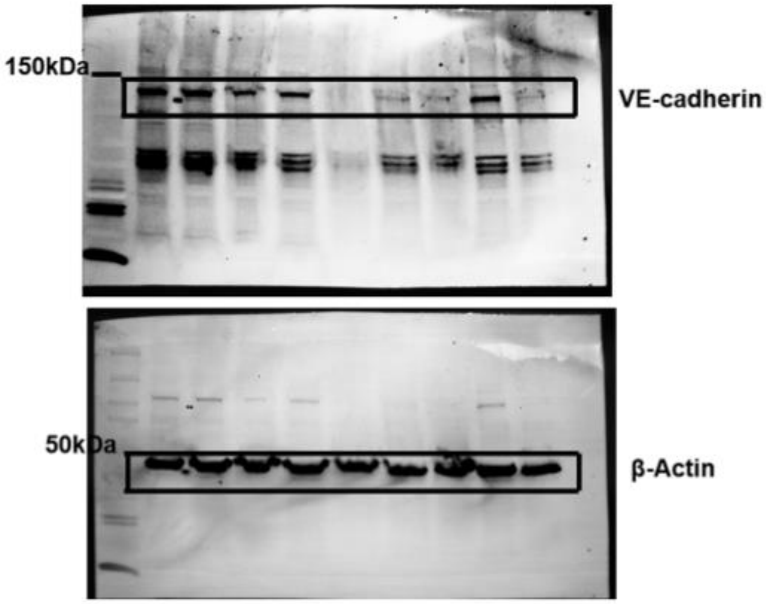

Supplementary Figure S6. Original full representative blots for main Fig. 5F.

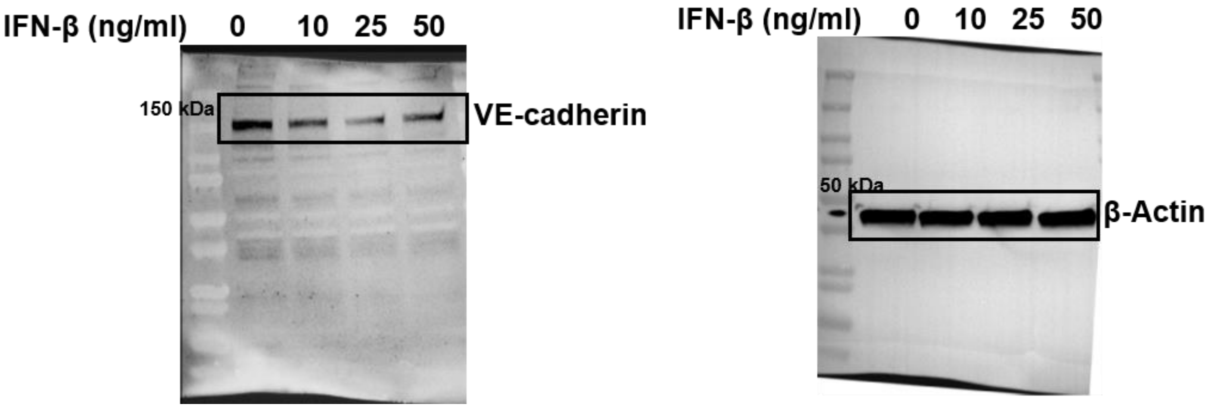

Supplementary Figure S7. Original full representative blots for main Fig. 6A.

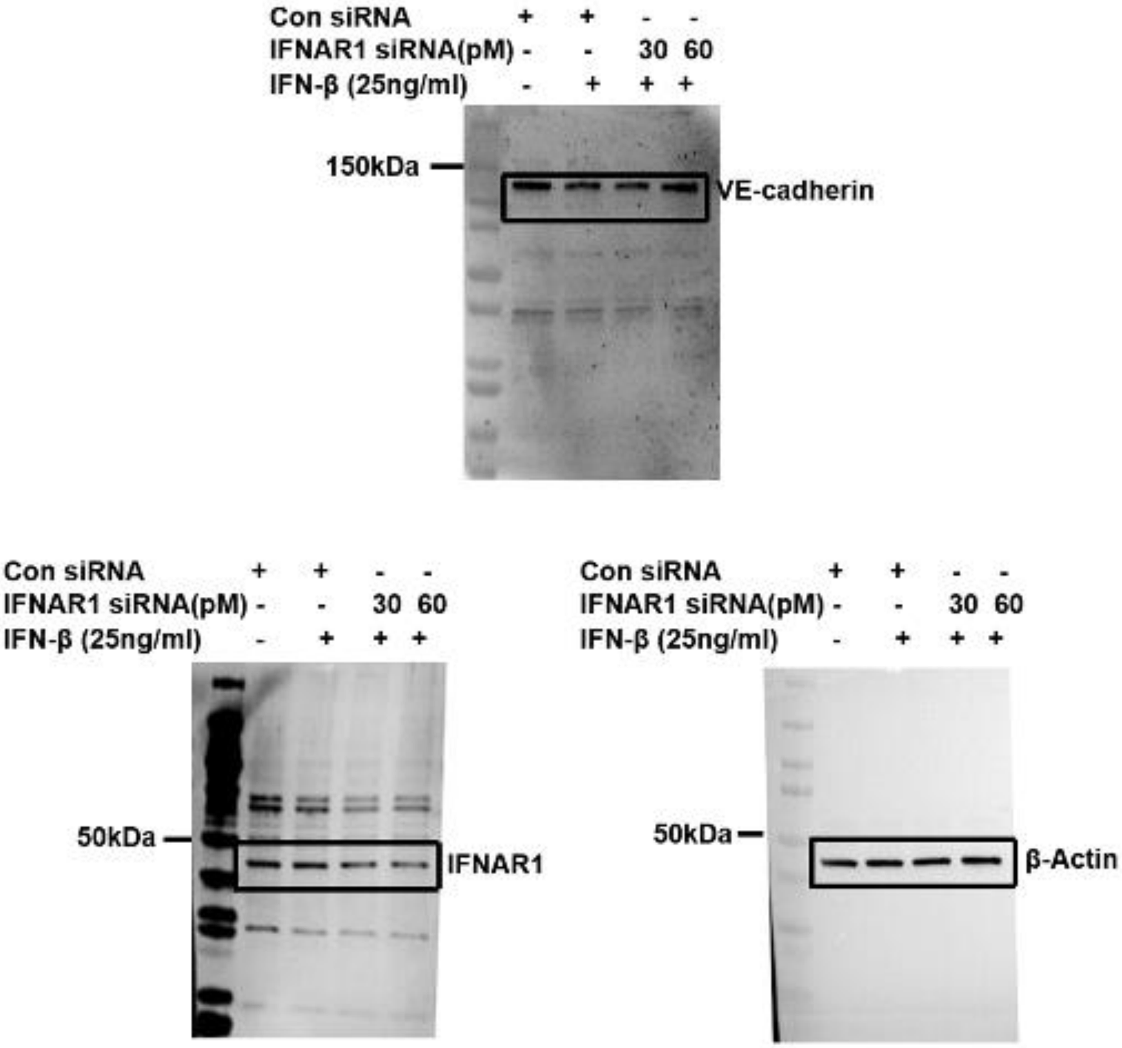

Supplementary Figure S8. Original full representative blots for main Fig. 7A.

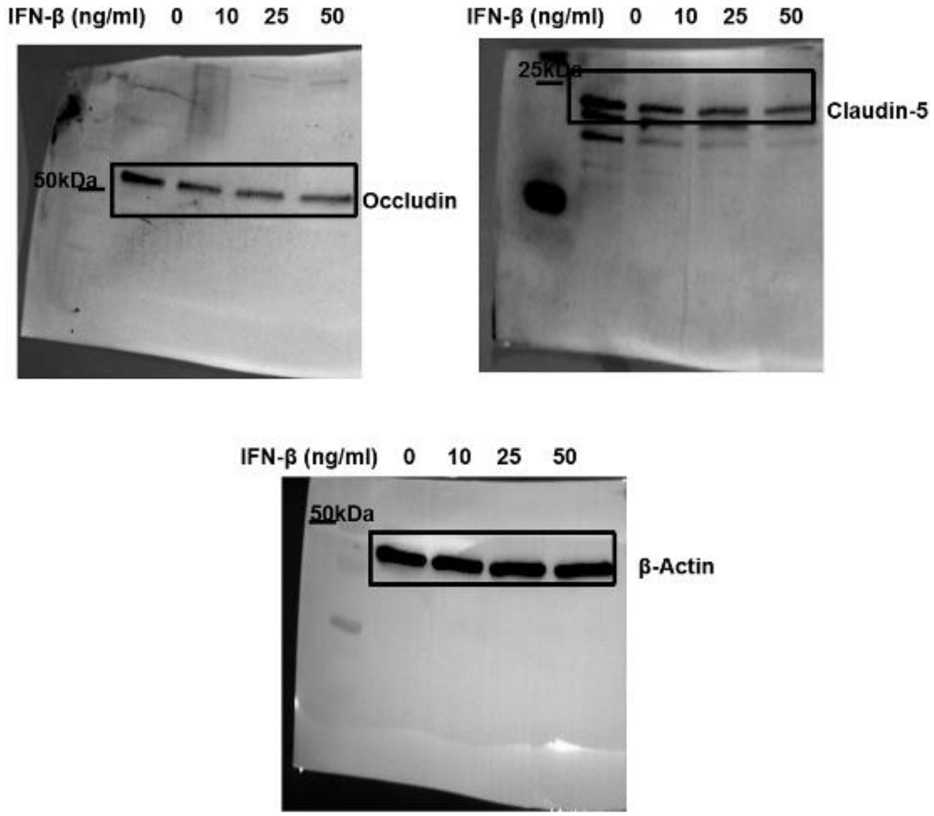

Supplementary Figure S9. Original full representative blots for main Fig. S4A.

Supplementary data analysis

Power analysis results for main Fig.1 and Fig.2

	Comparison	Within group variance	between group variance	power
**FITC dextran images cortex**	Control female1 yr vs. APP/PS1 female1 yr	0.0642	16.767	1.0000
	Control female 6 mon vs. APP/PS1 female 6 mon	0.1683	8.3465	1.0000
	APP/PS1 female 6 mon vs. APP/PS1 female1 yr	0.2324	1.1510	0.9998
**FITC dextran images hippocampus**	Control female 1 yr vs. APP/PS1 female1 yr	0.0014	12.5860	1.0000
	Control female 6 mon vs APP/PS1 female 6 mon	0.0005	0.0003	0.4140
	APP/PS1 female 6 mon vs. APP/PS1 female 1 yr	0.0014	12.4993	1.0000
